# Mouse models of multiple myeloma: technologic platforms and perspectives

**DOI:** 10.18632/oncotarget.24614

**Published:** 2018-04-13

**Authors:** Marco Rossi, Cirino Botta, Mariamena Arbitrio, Rosa Daniela Grembiale, Pierosandro Tagliaferri, Pierfrancesco Tassone

**Affiliations:** ^1^ Department of Experimental and Clinical Medicine, “Magna Graecia” University of Catanzaro, Catanzaro, Italy; ^2^ Department of Health Sciences, Magna Graecia University, Catanzaro, Italy; ^3^ Sbarro Institute for Cancer Research and Molecular Medicine, Center for Biotechnology, College of Science and Technology, Temple University, Philadelphia, PA, USA

**Keywords:** mouse models, multiple myeloma, SCID, SCID-hu, SCID-synth-hu

## Abstract

Murine models of human multiple myeloma (MM) are key tools for the study of disease biology as well as for investigation and selection of novel candidate therapeutics for clinical translation. In the last years, a variety of pre-clinical models have been generated to recapitulate a wide spectrum of biological features of MM. These systems range from spontaneous or transgenic models of murine MM, to subcutaneous or orthothopic xenografts of human MM cell lines in immune compromised animals, to platform allowing the engraftment of primary/bone marrow-dependent MM cells within a human bone marrow *milieu* to fully recapitulate human disease. Selecting the right model for specific pre-clinical research is essential for the successful completion of investigation. We here review recent and most known pre-clinical murine, transgenic and humanized models of MM, focusing on major advantages and/or weaknesses in the light of different research aims.

## INTRODUCTION

Multiple myeloma (MM) is characterized by the expansion of malignant plasma-cells (PCs) within a permissive bone marrow microenvironment (BMM) that promotes tumor cell survival and proliferation [1]. A preexisting premalignant condition, known as monoclonal gammopathy of undetermined significance (MGUS), is a common step of MM development [2]. The transition from MGUS to MM takes place by progressive mutational events accompanied by changes in the BMM [3, 4]. MM therapy has tremendously improved in the last few years together with the understanding of mechanisms promoting the onset and development of the disease [5]. Most of preclinical findings in MM research that translated into clinics as effective therapies have been developed by the use of a variety of *in vivo* models, which aim to recapitulate the disease and to provide insights on the interactions between MM cells and the surrounding microenvironment. Such models represent to date the basic tool to investigate and predict the effectiveness of novel therapeutic strategies. In this work, we will review the most relevant *in vivo* experimental platforms for the study of MM pathogenesis and for drug discovery.

## THE 5TMM MODEL: A MURINE BACKGROUND FOR MM DEVELOPMENT

**Figure 1:**
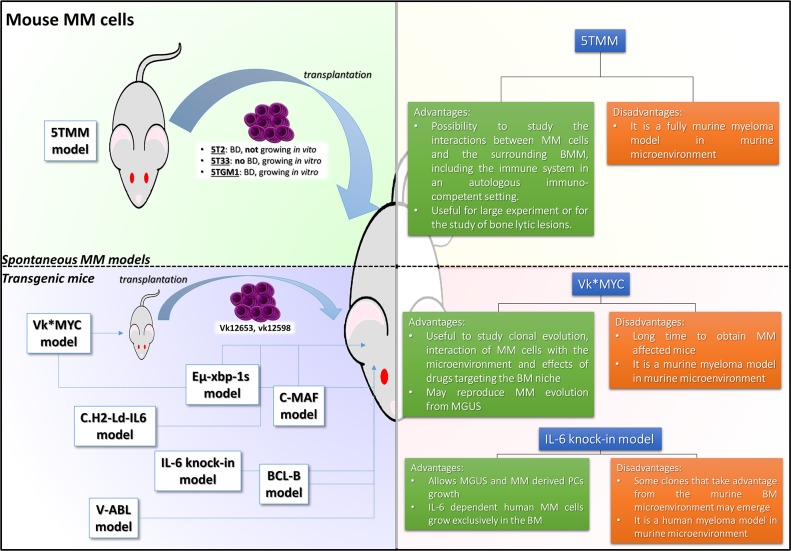
5TMM and TG mouse models of MM The pictures illustrate different strategies adopted to recapitulate MM disease. Models are divided according to their main characteristics: the green quadrant includes models of spontaneous mouse model of MM; the blue quadrant includes models of transgenic murine MM; each quadrant is accompanied by a table briefly describing the most relevant advantages and limits of each model. BD: bone disease; MM: multiple myeloma; BMM: bone marrow microenvironment

Specific mouse strains may spontaneously develop age-associated malignancies. Among them, C57BL/KalwRij are prone to develop B cell lymphoproliferative disorders, as approximately 80% of these mice carry a monoclonal component (MC), resembling human MGUS [6]. A very small fraction (0.5%) of mice progress to MM and Waldenstrom Macroglobulinemia. Mice spontaneously developing MM disease represent the original 5TMM model. Bone marrow (BM)-MM cells from these mice can be efficiently transplanted into syngeneic mice to easily reproduce the disease. Indeed, from the original 5TMM, several cell lines have been established, such as: 5T2 that reproduces different milestones of advanced disease including serum paraprotein production and lytic bone lesion formation; 5T33, more aggressive, with preferential dissemination to spleen and liver. It is therefore possible to derive mice carrying extensive or very limited skeleton damages, with malignant PC clones confined to the BM and spleen and with different growth patterns. In this model, cytogenetic abnormalities showed hyperdiploid features, with lower frequency of translocations as compared to human disease [7]. MM pathogenesis can be further evaluated by crossing mice with specific genetic backgrounds, such as recombinant activating gene 2 (-/-) (RAG-2 -/-) mice that lack T and B cell proper development [8]. These mice allow to study the interactions between MM cells and the surrounding BMM, including the immune system, and several findings such as the MM cells cross talk with BM stromal cells (BMSCs), the BM homing of malignant PCs and the overwhelming osteoclast (OCL) activity as the promoter of MM related bone disease (MMBD) are among the most relevant achievements of this model (Figure 1). Additionally, this model constitutes a suitable platform for drug discovery and has been largely used for *in vivo* evaluation of several new compounds, especially due to the significant number of animals bearing the same disease that may be easily produced, thus allowing the conduction of statistically relevant studies. However, the main limitation of this model resides in the exclusive murine genetic background that may limit the study of compounds targeting human specific targets.

All these points need to be considered in the translation process to the clinical setting.

## THE XENOGRAFT MODELS: PLATFORMS FOR NEW DRUGS SCREENING

Although the 5T model has been a milestone for the comprehension of MM pathogenesis, several investigators have tried to overcome the limit of “genetically murine myeloma”, in the aim of testing innovative drugs against human MM cells and their microenvironment. The earliest methods attempted to engraft human MM cells in an animal recipient relied on the injection either subcutaneously (s.c.) or systemically (intravenously, i.v. or intraperitoneally, i.p.) of MM cell lines in SCID and NOD/SCID mice. In these mice strains, engraftment of tumor cells is allowed by the lack of T and B cells (SCID and NOD/SCID) and reduced NK activity (NOD/SCID). The injection route is relevant in this system as the s.c. route will be followed by the growth of palpable tumors only in the injection site, while the systemic injection will give rise to disseminated involvement of different organs such as spleen, liver, lungs and bone marrow, depending on the cell line used [9–11]. In the s.c. model, the growth of MM cells can be easily measured by a caliper or ultrasound (that further allows the study of angiogenesis) until mouse death or sacrifice. Indeed, tumor volume and mouse survival are the main objective parameters to ascertain the effect of a specific drug. In the i.v. model, MM cell growth is diffuse in different organs and the burden of disease can be evaluated by standard imaging techniques (microCT/MRI/PET) [10]. Alternatively, MM cells are transduced to express a bioluminescent or fluorescent marker, thus allowing the detection of tumor cells by dedicated imaging devices. In both routes models, it is feasible to detect monoclonal component (MC) in the mouse sera as well as other biochemical markers (e.g. beta 2 microglobulin) that represent an indirect measurement of the disease burden. Both models have been developed in the aim to investigate new drugs and still represent the most common methods to study innovative drugs activity *in vivo* [12, 13].

The s.c. model has the advantage that the tumor can be easily monitored and measured during treatments. However, s.c. growth does not recapitulate the human disease due to several limitations. Specifically, in this model, MM cells proliferate in the absence of the peculiar BMM that sustains the expansion of malignant PCs and drives the development of bone damage and drug resistance. Additionally, MM cell lines used for engraftment are mainly derived from extramedullary sites such as pleural effusions or peripheral blood of advanced MM patients. These cells have lost the dependence from the BMM and have changed significantly their genetic background to a late stage disease- instead of intramedullary MM- type.

**Figure 2:**
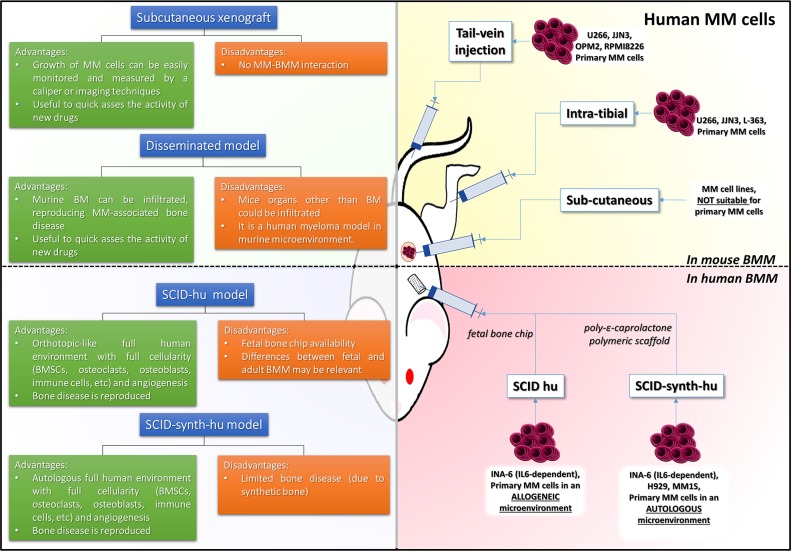
Xenograft and SCID models of MM The pictures illustrate different strategies to recapitulate human MM disease in mice. The yellow quadrant includes models where human MM cells are grown in murine bone marrow microenvironment; the red quadrant includes models where human MM cells are grown in human autologous or allogenic bone marrow microenvironment; each quadrant is accompanied by a table briefly describing the most relevant advantages and limits of each model. BD: bone disease; MM: multiple myeloma; BMM: bone marrow microenvironment

When considering the i.v. route, homing to the BM is also found, according to the specific type of MM cell line used. In these cases, the murine BM can be infiltrated with consequent bone damage as in the human MM. However, along the course of the disease, other mice organs host MM cells, reproducing an *in vivo* scenario commonly not observed in human disease. Thus, both models are very useful and easy-to-handle to quickly assess the activity of new cytotoxic drugs, but do not provide insights on the critical interactions between malignant PCs and their own BMM. This information is presently somewhat relevant to allow a successful translation in clinics of innovative drugs with selective mechanisms of action that in most cases involves the BMM. To overcome the relevant limitations of previous models, some attempts to develop new “humanized” models have been designed and made available (Figure 2). In these models, the basic idea is to reproduce the human BMM by implanting bone recipients in the flank of mice and then seeding them with fresh primary BM explanted cells or IL-6-dependent MM cell lines. The advantage of this approach is that MM cells grow in an orthotopic full human environment, where BMSCs support MM cell proliferation that in turn triggers osteoclasts (OCL) activity and suppresses bone apposition, thus reproducing the development of BD as observed in patients. Several variants of this model have been developed according to the type of the bone chip used (Figure 2).

### The SCID-hu model

**Figure 3:**
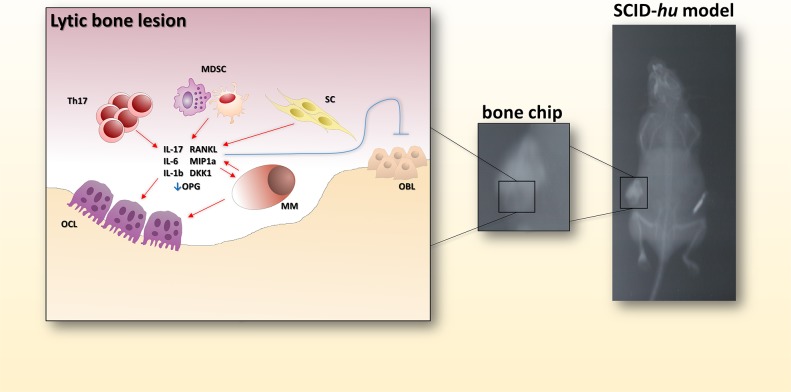
Bone-disease model The picture shows the radiologic evidence of a bone lytic lesion within a fetal bone chip. In the cartoon on the left, the main actors determining the bone resorption activity are reported. Th17: T helper 17 lymphocytes; MDSC: myeloid-derived suppressor cell; SC: stromal cell; OBL: osteoblast; MM: multiple myeloma cell; OCL: osteoclast.

In the SCID-hu model, a human fetal bone chip (FBC) is implanted in the recipient mouse. In this model [14], MM cell lines home exclusively to the human implant instead of murine bone. Furthermore, when a contralateral FBC was implanted, MM cells migrated from the previously injected FBC to the contralateral one, while sparing the murine bone and obviously all other murine normal tissues. Engrafted MM cells produced MC and IL-6, which correlated with the time from implantation and the tumor burden. Following this initial experience, primary PCs from MM patients were successfully engrafted into SCID-hu models [15]. MC was detected in mouse sera, while human bone sections showed increased OCL activity and neo-angiogenesis, resembling human disease (Figure 3). The FBC has been also demonstrated to be an excellent recipient for engraftment of IL-6 dependent MM cells (INA-6) [16]. These cells, in fact, are fully dependent on human BMSCs and/or human IL-6, which is a well recognized key factor and target for MM [17–28], and cannot be used for production of conventional s.c. xenografts since do not grow under murine IL-6 stimulation. On the other side, administration of human IL-6 induces mice cachexia and therefore these cells cannot be used *in vivo* without a recipient recapitulating the human bone *milieu*. In this model, extensive BM infiltration by INA-6 was paralleled by an increased beta 2 microglobulin in mice sera. The advantages of using the INA-6 implanted SCID-hu model are the unlimited high reproducibility of the data with a cell line that recapitulates more accurately the human disease, growing in an orthotopic environment. The latter is a crucial issue to study mechanistic pathways of myeloma-genesis and to ascertain the effects of new drugs against MM cells and BMM [16, 23, 26, 29–33].

Indeed, the use of these models allowed investigators to define the role of key signaling pathways or to investigate several new agents. For example, p38 mitogen activated kinase (MAPK) is constitutively activated in MM and contributes to tumor growth [34]. Abrogation of p38 signaling within MM cells attenuates OCL activity and restores bone apposition in a SCID-hu environment. The same model allowed demonstration of the anti-MM effects of thalidomide *in vivo* [35]. The potency of the model to study new drugs has been further tested by Tai et al., who showed that targeting Bruton tyrosine kinase (BTK) in MM cells and OCLs impairs MM growth and attenuates MMBD [38]. MM-dependent OCL activity is clearly reproducible in the SCID-hu model, where zoledronic acid, the most common bisphosphonate routinely used for MM supportive therapy, efficiently attenuates bone damage [35]. Osteoblasts (OBL) suppression and aberrant OBL/OCL crosstalk through Ephrin B2/B4 (EPHB2/B4) interaction has been also described within FBC [36]. The authors demonstrated that interfering with the EPH axis by EPH B2/B4 -FC molecules determined reversal of OBL suppression and reduced OCL levels in myeloma carrying SCID-hu mice.

Even if the SCID-hu model proved to be effective in studying MM pathogenesis as well as other plasma cell discrasias such as Waldenstrom's macroglobulinemia [37–39] and the activity of novel drugs, its major limitations rely on FBC availability for many institutions. Furthermore, fetal BMM does not exactly recapitulate adult BMM. Alternative methods have been developed in the aim to overcome these issues.

### The SCID-rab model

In the SCID-rab model human fetal bone chips are replaced by rabbit bones. MM cells engraft into the rabbit bone reproducing the human disease [40]. The SCID-rab model has been the backbone for studying the bone-building effects of proteasome inhibitor (PI) bortezomib, which are independent from anti-neoplastic effects of the PI. Indeed, when mice are exposed to PI, bone mineral density (BMD) increases in both myelomatous and non-myelomatous bones. These data represented the basis to further explore bone building activity of novel drugs within SCID-rab. In this model, [41] the effects of anti-DKK-1 mAb that was able to increase BMD in the presence of patient derived malignant PCs, has been evaluated. DKK-1 negatively regulates WNT signaling and OBL differentiation. By neutralizing DKK-1, OBL numbers were restored, while OCLs were significantly reduced. These findings were confirmed and implemented by testing a clinical grade anti DKK-1 Ab in the SCID-hu model [30], thus demonstrating the close relationship between the two models.

The SCID-rab model is a suitable model for preclinical investigation in MM, but it certainly retains its own limits as rabbit bone, although ontogenetically related, cannot be strictly compared to human bone. However, the development of bone chip based models unveiled the potentialities of this approach and prompted investigators to ameliorate this system.

### The SCID-synth-hu model

In an effort to reproduce human MM microenvironment in the mouse, synthetic scaffolds, closely resembling the trabecular structure of human bone were developed. A relevant option has been provided with the generation of the SCID-synth-hu model (Figure 2) [42]. It is based on a three-dimensional poly-ε-caprolactone polymeric scaffold (PS), featuring the human trabeculae, wherein MM cells/primary PCs and BMSCs can be successfully loaded. The PS is either cultured *in vitro* or implanted in the mouse flank as for FBC/rabbit chip. In this latter case, the recipient was then engrafted with cell populations from whole bone marrow aspirates directly *in vivo*. Treatment with i.p. bortezomib/dexametasone regimen led to dramatic decrease of MC in the mouse sera and clearance of MM cells within the retrieved scaffold. An intriguing preliminary finding was also the development of neo-angiogenesis in the MM coated PS [42]. Following-up this observation, Zhu et al. [43] demonstrated that bone marrow endothelial cells (BMECs) support MM cell growth, migration and BM homing within PS either *in vitro* and implanted in the mice. BMECs-MM cells interaction is mainly dependent on CD147, which is highly expressed by MM cells as compared to normal PCs and binds cyclophilin A (cyPA). cyPA is secreted by BMECs. CD147 blockade exerted potent anti MM activity as shown in the PS MM model. These data demonstrated that the PS is a powerful platform to recapitulate MM disease and to test a variety of innovative therapeutic approaches [29, 33, 42, 44–46]. Figure 2 summarizes the main characteristics of the most important models discussed in this section.

## SPECIAL CONSIDERATION ON THE USE OF NOD SCID GAMMA NULL MOUSE AS XENOGRAFT MODEL OF MM

The recently introduced NOD/SCID gamma null (NSG) mouse strain lacks IL-2 gamma chain, resulting in the absence of B/T/NK activity and severe impairment of antigen presenting cells and complement [47]. Some authors attained successful engraftment of I.V. injected U266 MM cells into NSG recipients following 2.4 gy irradiation (demonstrated to be unnecessary for tumor engraftment in subsequent reports) (Figure 2) [48]. U266 showed specific homing to murine BM, which is observed also with other MM cells such as OPM2, JJN3 and RPMI8226, and in contrast to NOD/SCID IV implants [10, 44, 49, 50]. Homing to the BM is reproducibly attained and classical features of MMBD can be observed with increased OCL activity, OBL reduction and lytic lesions across the skeleton [49]. However, it should be taken into account, that these models may present an extensive burden of extramedullary disease, thus mostly resembling very aggressive or late stage MM disease more than classical indolent myeloma. Again, the major limitation of this model is due to cell lines, that are better representative of a BMM-independent plasma cell leukemia than a MM.

Besides MM cells, patient derived malignant PCs have been engrafted into mice BM reproducing the human disease features. In this model, administration of zoledronic acid is effective in attenuating bone disease, while not affecting tumor burden. PIs such as carfilzomib dramatically reduced tumor burden and showed anabolic effects in MM implanted NSG mice, demonstrating that this model is an ideal platform for drug development and testing [49, 51]. A further refinement of NSG based MM models has been presented by others [52–54], where several MM cell lines, such as U266, L-363 and JJN3, or primary bone marrow cells from MM patients (both CD138 positive and negative fraction) have been directly implanted into NSG mice tibia (Figure 2). The advantages of intratibial implants are due to an accelerated and bone confined growth of MM, whose features are strictly related to the human disease. Interestingly, the disease spread over bone skeleton, and tumor burden could be easily determined by bioluminescent techniques in the presence of luminescent dye tagged MM cells and/or by MRI.

## TRANSGENIC MODELS

### IL-6 transgenic models

Early studies demonstrated that IL-6 is essential for MM cell growth *in vitro and in vivo* [55, 56], suggesting that this cytokine is the main supportive factor in mineral oil induced plasmacytomas (MOPCs) [57], which develop within the peritoneal cavity following the generation of inflammatory dependent granulomas [58]. Based on these premises, an IL-6 transgene (Tg) driven by the promoter of L^d^ gene of the major histocompatibility complex (H2-L^d^) was generated in B6 mice and showed the development of massive policlonal plasmacytomas. These tumors were transplanted in mineral oil treated BALB/c mice, where MC component of IgA isotype and clonal plasmacytomas were observed. These plasmacytomas carried t(12;15) and c-myc gene rearrangements. Interestingly, when IL-6 Tg is stabilized in BALB/c background mice through progressive backcrossing until N_20_, derived c.IL6 mice spontaneously developed monoclonal plasmacytomas or B cell lymphomas within lymphoid tissues. These tumors could be transplanted in mineral oil treated mice arising t(12;15)+ plasmacytomas [59]. These early studies were of relevance as they began to unveil the role of IL-6 dependence in MM growth and the importance of tumor microenvironment in promoting MM development in vivo*. C.H2-Ld-IL6* model is still the best reference model to study the pathophysiology of IL-6 in MM [60]. Further evidence of potential microenvironment dependence of MOPC tumors is provided by the derived MOPC315.BM cell line, which is obtained by consecutive i.v. passages of an established MOPC315 MM. Interestingly, these tumors shift from a s.c. growth form to BM selective expansion with extensive osteolytic disease and limited extramedullary involvement, resembling human MM [7].

### The v-ABL model

c-myc rearrangement in IL-6 driven plasmacytomas was not an isolated finding in early transgenic studies of MM. In the Eμ-v-*abl* model [61], the v-*abl* gene, which encodes for a constitutively active non-receptor tyrosine kinase, is juxtaposed with an IgH enhancer. The resulting Eμ-v-*abl* strongly promotes plasmacytoma development even in mineral oil free mice. In most cases a t(12;15) or other myc gene rearrangements could be identified, while double Tg Eμ-myc and Eμ-v-*abl* presented with an accelerated plasmacytoma onset (5-8 wks for double Tg as compared to 11-52 in Eμ-v-*abl*), thus confirming the putative role of c-MYC in MM pathophysiology. Acceleration of plasmacytoma onset can be attained by crossing the Eμ-v-*abl* mice with either Bim^-^/^-^, BCL2 or MCL-1 Tg mice [62]. The lack of pro- apoptotic signals or the enriched survival signals carried by crossed Tg facilitated MYC driven plasmacytoma-genesis by increasing PC frequency in normal mice and increased cyclin D expression. The synergistic effects of MYC and *abl* can be due to the activation of relevant pro-survival and proliferation signaling pathways such as RAS and PI3K dependent pathways. This synergy seems to be selective for tumorigenesis in PC lineage rather than lymphocytes, although the mechanisms of such selectivity remain not clarified.

### The Vk^*^MYC model

In the mineral oil/transgene induced plasmacytomas discussed so far, tumors develop in the peritoneum, lymphnodes, spleen and more rarely BM. Therefore, these models resemble at best extramedullary MM cases and do not recapitulate the human disease. As discussed, a common element of these models is the rearrangement of MYC gene that drives tumor onset and development. The Vk^*^MYC model [63] aims to overcome these limitations and to approximate the generated mouse tumor to human MM (Figure 1). The Vk^*^MYC is structured with a V-kappa exon sequence splicing in frame with the human MYC locus. The Tg harbors a stop codon within V-kappa exon that generates a DGYW motif. This motif is targeted by somatic hyper-mutation machinery (SHM), which can randomly revert the stop codon and promote MYC expression. In this way, MYC becomes activated along GC reaction, preferentially affecting PC differentiation as light chain regulatory sequences allow for SHM activity. Low proliferating monoclonal PCs accumulate into Vk^*^MYC mice, along their life, within BM and secondary lymphoid organs, and resemble the feature of MGUS/MM. Antibody production is monoclonal in most cases. Bi- or tri-clonal paraprotein occurs to a lesser extent, indicating the occurrence of different clonal PCs. Clonal PCs are transplanted into syngenic mice recapitulating MGUS with corresponding MC spikes in recipient mice. The range of BM PC infiltration in Vk^*^MYC mice encompasses true MGUS disease up to overt MM. These mice have on average lower hemoglobin levels, reduced BMD and MM like kidney damage, thus resembling the human disease. The peculiarity of this model is that the tumor recapitulates the slow development and progression of MM with only 1/3 of the mice that evolve towards an extramedullary plasmacytoma as opposite to previously discussed models. The “smoldering” phenotype of the model switches to a more aggressive disease when BM PCs are serially transplanted in congenic mice [64]. The Vk^*^MYC has been used to evaluate the activity of anti-MM drugs [64]. PIs and ImiDs and innovative drugs such as HDAC inhibitors are effective drugs in the model, while standard chemotherapy lacks relevant activity, resembling the human disease. The model is also suitable to study and develop novel combo therapies. Another peculiarity of the Vk^*^MYC model is the reproduction of the existing cross talk between clonal PCs and surrounding microenvironment. This distinctive feature proves to be very helpful to study clonal dynamics of the disease. As for other tumor types, the current hypothesis of MM onset and development is based on the existence of competition among different clones at diagnosis and along treatment, that drives towards indolent or more aggressive disease courses [4]. Indeed, the Vk^*^MYC models allows the study of the evolution of distinct clones in the same mouse along time [65]. These clones are identified after transplant in congenic wild type or Vk^*^MYC mice to evaluate their behavior within transplanted mice. Interestingly, transplanted clones can compete out the preexisting tumor clones within the recipient mice, coexist with them or even support these clones to proliferate. A similar pattern is observed also in Vk^*^MYC mice undergoing treatment which by itself creates clonal selection. These different courses resemble the human disease and can be observed also in MM at diagnosis and during subsequent relapses [4]. The model highlights the complex role of MYC in MM onset and development. Several findings indicate that MYC is involved in MGUS to MM transition [66], while MYC inhibitors efficiently promote MM cell death *in vitro* [67]. If we consider that MYC activation in MM is sustained mainly by secondary translocations not involving Ig Locus and can be present in the early phases of MM development [68], it becomes further evident the relevance of the Vk^*^MYC model to study MM pathogenesis.

### The XBP-1 model

Unfolded protein response (UPR) has a recognized role in PC differentiation and function [69]. Among regulators of UPR, XBP-1 has been investigated [69–71]. XBP-1 is required for PC differentiation as it is rapidly upregulated in B cells upon PC differentiating stimuli, whereas mice lacking XBP-1 retain normal B cell activity but show very low level of antibodies in the absence of PC differentiation. Based on these premises, a Eμ-XBP-1 Tg mouse, with a prominent expression of Tg product in lymphoid organs (spleen, lymphnodes, bone marrow and thymus) has been generated [72]. By 40 wks of age, transgenic mice developed M spike detectable in sera, antibody based cast nephropathy and bone marrow PC infiltrate <10%. At 14-24 months of age, 1/3 of mice developed MM, showing >10% bone marrow PCs and bone lytic lesions. A human MM like gene signature was identified in Eμ-XBP-1 mice including over-expression of Cyclin D1 and MAF. The Eμ-XBP-1 Tg mouse represents a suitable platform to study the consequences of targeting UPR modulators in MM. gp96 is a ER related chaperone, that is strictly related to XBP-1 activity and found upregulated in MM patients [73]. gp96 deletion within Eμ-XBP-1 Tg mice attenuates MM disease, further linking UPR and MM development in this model.

### The c-MAF model

c-MAF proto-oncogene is found over-expressed in MM in the presence of t(14;16) [74]. On this basis, a TG mouse carrying the Eμ-IgH-c-MAF vector that allowed expression of the TG in B cell lineage has been generated. The same group previously demonstrated that c-MAF upregulation in T cell compartment led to development of T cell lymphomas [75]. Similarly, B cell compartment restricted over-expression of c-MAF favors B cell lymphomas in aged mice (>50 weeks of age) with typical features: a clonal M spike with hyper-gammaglobulinemia, increased PCs in the bone marrow and cast nephropathy. Interestingly, tumor cells were B220+CD138+CD21+CD23+IgM+IgD-, thus resembling a plasma-cytic or plasma-blastic lymphoma. C-MAF targets such as Ccnd2 and Itgb7 were consistently elevated as expected, while the transcription factors Blimp-1 and XBP-1, which control PC differentiation were increased. In contrast, the main negative regulator of PC development, PAX-5, was downregulated in these mice. Overall, these data confirm the relevance of c-MAF in the development of PC tumors as described in MM patients. However, c-MAF TG mice do not recapitulate MM disease as the behavior of these tumors is more similar to PC lymphomas. Therefore, the mechanism of MM development in C-MAF carrying tumors must be further elucitated.

## THE BCL-2 FAMILY PROTEINS TG MODELS

BCL-2 family members exert potent anti-apoptotic activity supporting PC survival upon microenvironment stimuli [76]. Among them, BCL-XL and Bcl-B were studied in TG models of PC tumors. A BCL-XL TG was developed with a K chain enhancer (KE) and promoter (Vk21, KP) [77]. BCL-XL TG developed hyper-gammaglobulinemia, extramedullary PC foci together with a MM like cast nephropathy. However, no M spikes were found. When backcrossed with Eμ-c-MYC mouse, the double TG mice developed aggressive B cell tumors, with severe lymphocytosis, hypergammaglobulinemia and a fatal outcome by 5.5 weeks of age. While multiple extramedullary PC loci are generated, BM PC counts are not increased. However, lytic disease is observed in the long bones of sacrificed mice. Genetic features of malignant PCs of double TG mice were analyzed in a second report [78], showing the presence of several translocations, deletions and insertions. The most frequent aberrant chromosomes were 12 and 16. Among dysregulated genes, cyclin group genes were abnormally expressed as compared to normal PCs.

Bcl-B is a BCL-2 family member, whose role in MM pathogenesis has been previously overlooked [79]. Indeed, Bcl-B expression is increased in MM cell lines and malignant PCs from patients as compared to MGUS and healthy subjects. TG Eμ-IgH-Bcl-B mice showed a significantly shorter survival as compared to WT mice. They developed progressive BM plasmocytosis, anemia and M spike in the sera. Interestingly, IgG2b was the only isotype expressed in TG mice, a feature unexplained by investigators [79]. GEP was carried out on BM PCs from these mice unveiling the expression of typical human MM associated genes, driving PC survival and proliferation, and triggering bone resorption [79]. BM PCs from TG mice were transplantable in syngeneic mice reproducing the disease in the recipient. BM plasmocytosis in TG mice was reduced by treatment with standard anti MM drugs (bortezomib and melphalan). These data indicated that TG Eμ-IgH-Bcl-B mice recapitulate the human MM disease more stringently than BCL-XL TG and can be used as a platform for testing new drugs.

## THE “IL-6 KNOCK IN” HUMANIZED MOUSE MODEL

The research of mouse models that could recapitulate human hematopoiesis and hematological malignancies has followed parallel steps along the last 20 years. However, the experiences in SCID-NOD/SCID environment proved to be suboptimal for reproducing human hematopoiesis [80]. On this regard, a major improvement has been achieved with the development of humanized mice where human cytokine genes were knocked in to replace the corresponding mouse cytokine counterpart [80, 81]. This approach allows human hematopoietic cell growth upon more physiological stimuli as the expression of the selected human cytokine is tissue specific and finely regulated as opposite to the insertion of a TG or the administration of an exogenous cytokine. Based on these premises, a Rag2-/- IL2R -/- mouse was generated where human genes for M-CSF, GM-CSF, IL-3 and TPO were knocked in the corresponding mouse loci. This mouse (MITRG) allowed easier engraftment of human hematopoietic cells derived from peripheral blood or BM [81] and development of innate immune system cells (Figure 1). The latter is a very relevant feature of this model as human innate immune system plays a prominent role in the study of tumor growth in the context of chronic inflammatory stimuli. Indeed, Das et al. [82] knocked in IL-6 human gene in the MITRG mouse, followed by injection of INA-6 MM cell line or patient derived malignant PCs into the bone. In this system, MM PCs nicely grew within mouse BM, without showing contralateral bone and splenic proliferation, meaning that tumor cells followed a growth path that was limited to the bone as in the human disease. Interestingly, either CD138+ or CD138- BM cell fraction expanded into MITGR BM. Tumor cell fraction obtained from grafted mice was able to reproduce the disease upon transplantation in tumor free mice. Transplanted tumor showed better engraftment and growth after CD3 T cell depletion from primary tumor to attenuate xenograft versus host disease. MITGR mice proved to be an ideal recipient to allow malignant PC proliferation from MGUS and smoldering MM [82]. Comparison of genomic analysis by whole-exome sequencing between xenograft tumor cells and parental MM demonstrated similar LOH and CNAs patterns. This finding is very relevant when we consider the possibility to evaluate the evolution of distinct sub-clones within the mouse model. Indeed, an ideal model should preserve the clonal distribution observed in the parental tumor cells derived from the patient. However, even in this model, genomic changes could be detected in the xenografted tumors. As authors argued, the changes were likely due to the emergence of minor sub-clonal fractions that take a proliferation advantage in the xenograft BM microenvironment [82]. Table 1 summarizes the different TG models discussed in this section.

**Table 1:** Transgenic murine models of MM

TG Model	TG	Genomic aberrations	Growth Pattern
***C.H2-Ld-IL6***	L^d^-IL-6	T(12,15); c-MYC rearrang	E.M. monoclonal plasmacytomas/B cell lymphomas
***v-ABL***	Eμ-v-abl	T(12,15); c-MYC rearrang	E.M. monoclonal plasmacytomas
Vk^*^MYC	V-k exon-c-MYC	SHM dependent c-MYC expression in CD138+ PCs	I.M. MGUS/MM
***XBP-1***	Eμ-xbp1	Cyclin D1; MAF upreg.	I.M. MGUS/MM
***c-MAF***	Eμ-IgH-c-MAF	X-BP1, Blimp-1 upreg.; PAX-5 dowreg.	Plasmablastic/plasmacytic lymphomas
***Bcl-B***	Eμ-IgH-Bcl-B	IGF-1, IL-6, c-MYC, X-BP1, IRF-4 upreg.	I.M. MM
***IL-6 Knock in***	Rag2 -/- IL2R -/-/hIL-3-MCSF-GMCSF-TPO-IL-6 knock ins	Parental MM cell aberrations	Recipient of MGUS/sMM/MM PCs I.M. growth pattern

Table 1 illustrates the main TG models discussed in the text together with the most relevant genomic aberrations detected in TG mice and the pattern of proliferation of malignant PCs (see text for further details). Abbreviations: rearrang.: genomic rearrangement; upreg.: upregulated gene; downreg: downregulated gene; SHM: somatic hypermutation machinery; I.M.: intramedullary; E.M.: extramedullary.

## IMMUNOTHERAPY: THE NEW CHALLENGE FOR MURINE MODELS

In recent years, we observed a paradigm shift in the treatment of different malignancies with the increasing introduction into the clinical practice of novel agents or innovative strategies aimed to improve patients’ immune response against cancer cells [83–89]. Specifically, the discovery of immune checkpoints and the rapid development of inhibitory monoclonal antibodies to these molecules raised the question on the best suitable model to choose for the study of new immune-modulatory agents. In this view, several immunocompetent mouse models of MM, including the 5TMM and vk^*^MYC, have been widely used to investigate the role of different immunosuppressive cell populations and the activity of checkpoints inhibitors such as anti-PD1 mAbs [90–95]. However, further efforts are required to generate the “ideal” model for cancer immunotherapy, i.e. mice bearing a fully competent immune system able to mount an effective anti-cancer response [96]. The SCID-hu and the SCID-synth-hu represent a strong improvement in this direction, especially taking into account that immune populations, such as dendritic cells, have been reported to be present and functionally active in the latter model [97].

## CONCLUDING REMARKS AND PERSPECTIVES

*In vivo* models of MM have increased the understanding of the disease complexity. On a historical point of view these models have evolved with the major aim of better defining the complexity of the cross-talk between tumor cells and their BMM and also to provide predictive tools for investigation of new agents. The recent interest in the discovery of agents capable of targeting and modulate regulatory networks driven by non-coding agents [98, 99] has further underscored the need of more reliable models of human MM. On these bases, selecting the right model for each specific pre-clinical research is an essential point for the successful completion of investigation. In general, if we recollect the different systems, it is clear that humanized mouse models likely represent the best platform for future studies. Among others, two major breakthrough findings have recently emerged in the pathogenic and therapeutic scenario of MM: the clonal evolution [4] and the immunotherapy [100, 101]. MM models that allow recapitulation of the clonal competition for investigation of novel biological agents as well as those that allow the design and evaluation of immunotherapeutic strategies within an appropriate microenvironment, will likely provide the most relevant platforms and impact in future translational studies on MM.
